# Supplementary material for: DNA polymerase α-primase can function as a translesion DNA polymerase
Source: bioRxiv. 2025 Jul 2:2025.07.02.662785. Preprint. [Version 1] doi: 10.1101/2025.07.02.662785 (PMC12236624; doi:10.1101/2025.07.02.662785)
Supplement: 1 [file NIHPP2025.07.02.662785v1-supplement-1.pdf]

bioRxiv preprint doi: https://doi.org/10.1101/2025.07.02.662785; this version posted July 2, 2025. The copyright holder for this preprint (which was not certified by peer review) is the author/funder, who has granted bioRxiv a license to display the preprint in perpetuity. It is made available under aCC-BY 4.0 International license.

Supplemental Information

Table S1: Sequences (5'=>3') of oligonucleotides used in this study

**Pr1**	GTCTCGAGCCCATCCTTCCACTTCCCAACCCTCACC
**Std1**	GTCTCGAGCCCATCCTTCCACTTCCCAACCCTCACCTTTAAAAAAAAAAAAAAAAAAAAA AAAAAAAAAAAAAAAAAACACTCTCCAATTCTCACTTCCTACCACATCCCT
**Std2**	GTCTCGAGCCCATCCTTCCACTTCCCAACCCTCACCTTTAAAAAAAAAAAAAAAAAAAAA AAAAAAAAAAAAAAAAAACACTCTCCAATTCTCACTTCCTACCACATCCCTATTCTAACCA CATTAATCTACACCTATATATATATATCGCGACTGAG
**Std3**	GTCTCGAGCCCATCCTTCCACTTCCCAACCCTCACCCACTCTCCAATTCTCACTT
**Std4**	GTCTCGAGCCCATCCTTCCACTTCCCAACCCTCACCCACTCTCCAATTCTCACTTTCTAC CACATCCCTATTCTAACCACATTA
**LeadNL-long**	CTCAGTCGCGATATATATATATAGGTGTAGATTAATGTGGTTAGAATAGGGATGTGG TAGGAAGTGAGAATTGGAGAGTGTTTTTTTTTTTTTTTTTTTTTTTTTTTTTTTTTTTT TTTTTAAAGGTGAGGGTTGGGAAGTGGAAGGATGGGCTCGAGACGTTTTTTTTT TTTTTTTTTTTTTTTTTTTTTTTTTTTTTTT
**Lead-Tg**	CTCAGTCGCGATATATATATATAGGTGTAGATTAATGTGGTTAGAA(**Tg**)AGGGATGT GGTAGGAAGTGAGAATTGGAGAGTGTTTTTTTTTTTTTTTTTTTTTTTTTTTTTTTTTT TTTTTTTAAAGGTGAGGGTTGGGAAGTGGAAGGATGGGCTCGAGACGTTTTTTTT TTTTTTTTTTTTTTTTTTTTTTTTTTTTTTTTT
**Lead-CPD**	CTCAGTCGCGATATATATATATAGGTGTAGATTAATGTGGTTAGA(**CPD**)AGGGATGT GGTAGGAAGTGAGAATTGGAGAGTGTTTTTTTTTTTTTTTTTTTTTTTTTTTTTTTTTT TTTTTTTAAAGGTGAGGGTTGGGAAGTGGAAGGATGGGCTCGAGACGTTTTTTTT TTTTTTTTTTTTTTTTTTTTTTTTTTTTTTTTT
**LeadNL-short**	TAATGTGGTTAGAATAGGGATGTGGTAGGAAGTGAGAATTGGAGAGTGGGTGAG GGTTGGGAAGTGGAAGGATGGGCTCGAGACTTT
**Lead-Ab**	TAATGTGGTTAGAATAGGGATGTGGTAG(**Ab**)AAGTGAGAATTGGAGAGTGGGTG AGGGTTGGGAAGTGGAAGGATGGGCTCGAGACTTT
**Lead-8-oxoG**	TAATGTGGTTAGAATAGGGATGTGGTAG(**8oxoG**)AAGTGAGAATTGGAGAGTGG GTGAGGGTTGGGAAGTGGAAGGATGGGCTCGAGACTTT
**D25 DNA Primer**	GTTAAAGGCCGCTTTTGCGGGATCG
**R25 RNA Primer**	rGrUrUrArArArGrGrCrCrGrCrUrUrUrUrGrCrGrGrGrArUrCrG

bioRxiv preprint doi: https://doi.org/10.1101/2025.07.02.662785; this version posted July 2, 2025. The copyright holder for this preprint (which was not certified by peer review) is the author/funder, who has granted bioRxiv a license to display the preprint in perpetuity. It is made available under aCC-BY 4.0 International license.

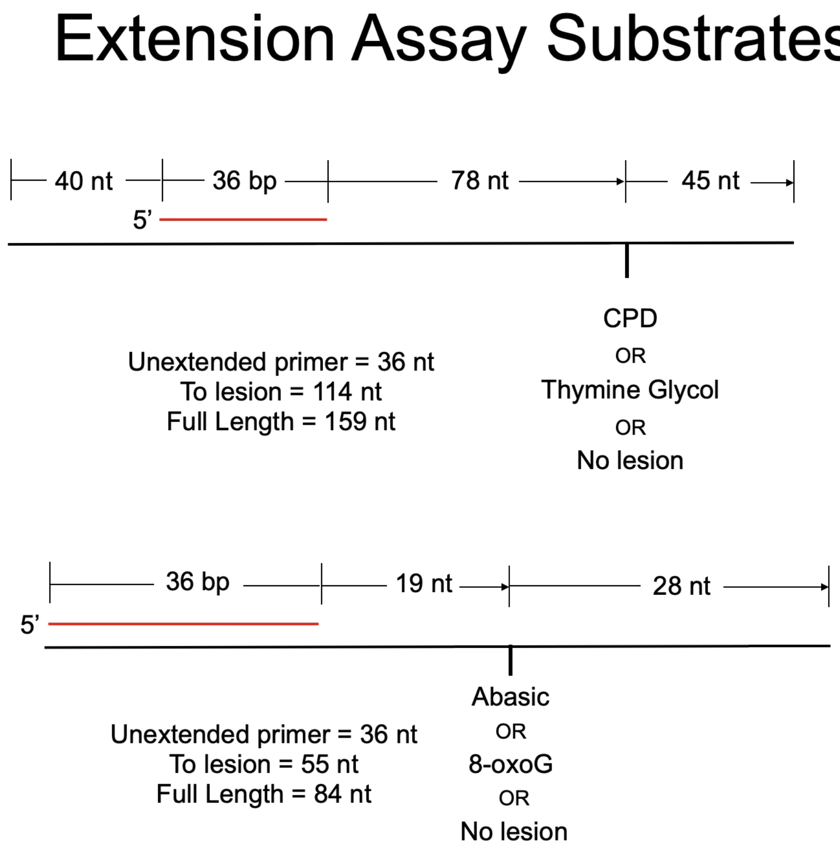

Figure S1: Diagrams for primer extension substrates. Details of the sizes for each section of the substrates used in our assay along with lengths of benchmark products. The primer, in red, is 5' end labeled with ^{32}P-ATP.

bioRxiv preprint doi: https://doi.org/10.1101/2025.07.02.662785; this version posted July 2, 2025. The copyright holder for this preprint (which was not certified by peer review) is the author/funder, who has granted bioRxiv a license to display the preprint in perpetuity. It is made available under aCC-BY 4.0 International license.

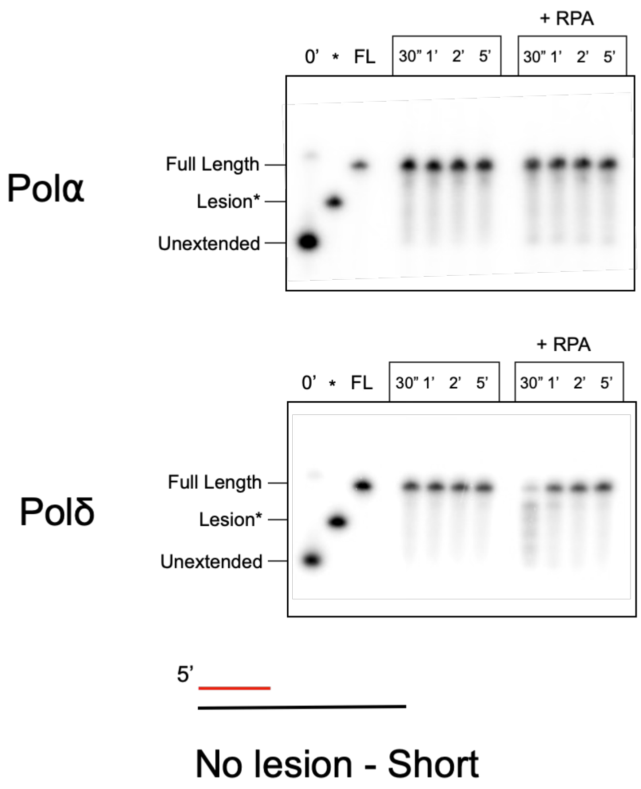

Figure S2: Primer extension assays on the short no lesion substrate. Extension products generated by Polα or Polδ with and without RPA. The * indicates products of a size corresponding to extension up to a lesion in the equivalent lesion substrates. This is included as a reference to show no pausing occurs at this location when no lesion is present. The ^{32}P-ATP end-labeled primer is indicated in red.

bioRxiv preprint doi: https://doi.org/10.1101/2025.07.02.662785; this version posted July 2, 2025. The copyright holder for this preprint (which was not certified by peer review) is the author/funder, who has granted bioRxiv a license to display the preprint in perpetuity. It is made available under aCC-BY 4.0 International license.

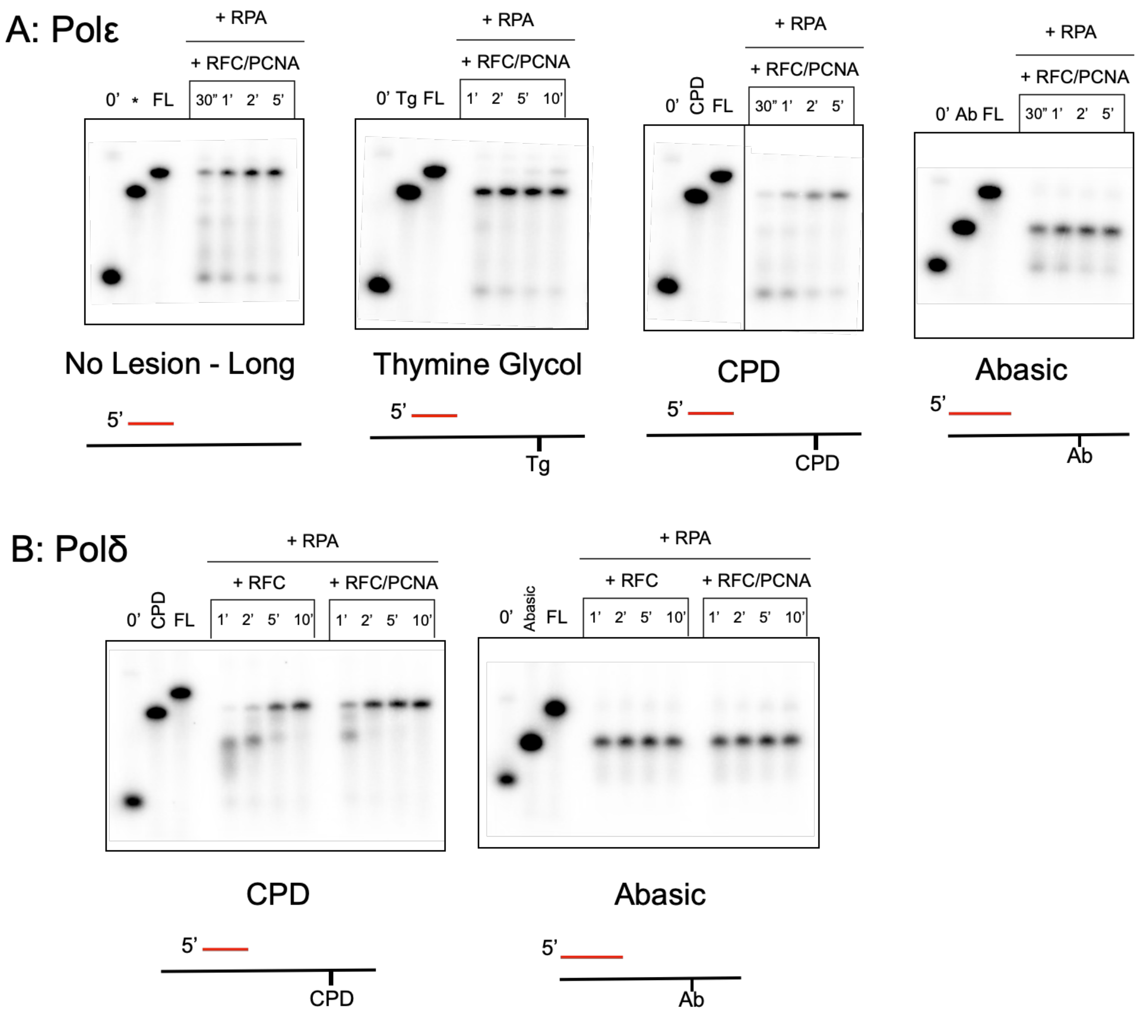

Figure S3: Impact of RFC +/- PCNA on extension and lesion bypass. Extension products are shown for assay with Polε or Polδ on the indicated substrate. In all cases, RPA was first loaded followed by initiation of extension by addition of polymerase, RFC, PCNA, ATP, and dNTPs. The * lane in no lesion substrate experiments indicates the size of products where stalling at a lesion would occur in the lesion carrying substrates. Red primers are ^{32}P-ATP end-labeled.

bioRxiv preprint doi: https://doi.org/10.1101/2025.07.02.662785; this version posted July 2, 2025. The copyright holder for this preprint (which was not certified by peer review) is the author/funder, who has granted bioRxiv a license to display the preprint in perpetuity. It is made available under aCC-BY 4.0 International license.

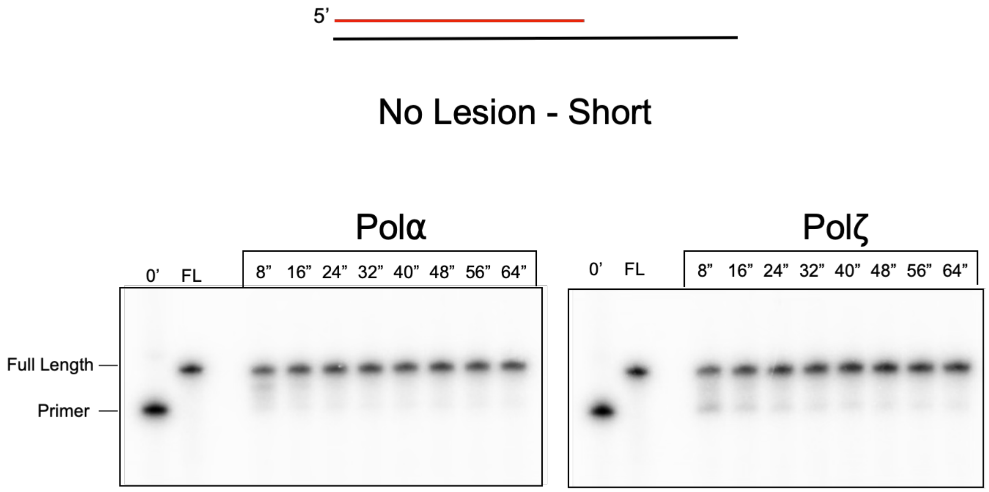

Figure S4: Primer extension on the short no lesion control substrate. Extension products from assays with Polα or Polζ at indicated timepoints. Unlike previous experiments, these are primed one nucleotide upstream of the site of lesions in parallel substrates. This illustrates that the 28 nucleotides of extension is completed very quickly. The red primer strand is ^{32}P-ATP end-labeled.
